# Evolution of anti-modified protein antibody responses can be driven by consecutive exposure to different post-translational modifications

**DOI:** 10.1186/s13075-021-02687-5

**Published:** 2021-12-08

**Authors:** M. Volkov, A. S. B. Kampstra, K. A. van Schie, A. Kawakami, M. Tamai, S. Kawashiri, T. Maeda, T. W. J. Huizinga, R. E. M. Toes, D. van der Woude

**Affiliations:** 1 Department of Rheumatology, Leiden University Medical Center, PO Box 9600, Albinusdreef 2, 2300 RC Leiden, The Netherlands; 2 Department of Immunology and Rheumatology, Nagasaki University Graduate School of Biomedical Sciences, Nagasaki, Japan; 3 Department of Community Medicine, Nagasaki University Graduate School of Biomedical Sciences, Nagasaki, Japan; 4 Department of General Medicine, Nagasaki University Graduate School of Biomedical Sciences, Nagasaki, Japan

**Keywords:** Rheumatoid arthritis, Anti-citrullinated protein antibodies (ACPA), Anti-carbamylated protein antibodies (Anti-CarP), Anti-acetylated protein antibodies, (AAPA) Anti-modified protein antibodies (AMPA)

## Abstract

**Background:**

Besides anti-citrullinated protein antibodies (ACPA), rheumatoid arthritis patients (RA) often display autoantibody reactivities against other post-translationally modified (PTM) proteins, more specifically carbamylated and acetylated proteins. Immunizing mice with one particular PTM results in an anti-modified protein antibody (AMPA) response recognizing different PTM-antigens. Furthermore, human AMPA, isolated based on their reactivity to one PTM, cross-react with other PTMs. However, it is unclear whether the AMPA-reactivity profile is “fixed” in time or whether consecutive exposure to different PTMs can shape the evolving AMPA response towards a particular PTM.

**Methods:**

Longitudinally collected serum samples of 8 human individuals at risk of RA and 5 with early RA were tested with ELISA, and titers were analyzed to investigate the evolution of the AMPA responses over time. Mice (13 per immunization group in total) were immunized with acetylated (or carbamylated) protein (ovalbumin) twice or cross-immunized with an acetylated and then a carbamylated protein (or vice versa) and their serum was analyzed for AMPA responses.

**Results:**

Human data illustrated dynamic changes in AMPA-reactivity profiles in both individuals at risk of RA and in early RA patients. Mice immunized with either solely acetylated or carbamylated ovalbumin (AcOVA or CaOVA) developed reactivity against both acetylated and carbamylated antigens. Irrespective of the PTM-antigen used for the first immunization, a booster immunization with an antigen bearing the other PTM resulted in increased titers to the second/booster PTM. Furthermore, cross-immunization skewed the overall AMPA-response profile towards a relatively higher reactivity against the “booster” PTM.

**Conclusions:**

The relationship between different reactivities within the AMPA response is dynamic. The initial exposure to a PTM-antigen induces cross-reactive responses that can be boosted by an antigen bearing this or other PTMs, indicating the formation of cross-reactive immunological memory. Upon subsequent exposure to an antigen bearing another type of PTM, the overall reactivity pattern can be skewed towards better recognition of the later encountered PTM. These data might explain temporal differences in the AMPA-response profile and point to the possibility that the PTM responsible for the initiation of the AMPA response may differ from the PTM predominantly recognized later in time.

**Supplementary Information:**

The online version contains supplementary material available at 10.1186/s13075-021-02687-5.

## Background

In the last few decades, many insights have been gained about the role of autoantibody responses in the pathophysiology of rheumatoid arthritis (RA). Anti-citrullinated protein antibodies (ACPA) have provided a new angle on disease development and become a useful diagnostic biomarker [1, 2].

Although the exact role of the ACPA response in disease development is not yet clear, considerable insights have been obtained about the nature of this response and its evolution before and after disease onset. Interestingly, ACPA can be present for years in individuals without symptoms and the presence of ACPA alone does not necessarily lead to disease manifestations; these individuals can revert to seronegativity with time [3]. In the individuals that will develop RA, the ACPA response matures prior to the disease onset as shown by an increase in ACPA levels, epitope spreading, broader isotype usage and partial avidity maturation [4, 5]. Upon disease onset, the ACPA response may undergo another change, as active treatment with disease-modifying anti-rheumatic drugs (DMARDs) can lead to a decrease of autoantibody levels, although rarely results in autoantibody serum levels turning negative [6, 7]. Overall, the evolution of the ACPA response before and throughout the disease appears to be a complex phenomenon, as its immunological underpinnings remains to be understood.

In the last few years, it has become evident that citrullination is not the only post-translational modification (PTM) recognized by autoantibodies in RA patients, as anti-carbamylated protein antibodies (anti-CarP), anti-acetylated protein antibodies (AAPA), and anti-malondialdehyde-acetaldehyde antibodies (anti-MAA) were discovered in the serum of RA patients [8–10]. Importantly, although the anti-MAA response is not specific for RA, these autoantibody responses tend to co-exist with ACPA. Moreover, PTMs recognized by ACPA, anti-CarP, and AAPA (i.e., citrulline, homocitrulline and acetyllysine) share structural similarities, while homocitrulline and acetyllysine also share the amino acid of origin—lysine. Cross-reactivity can be observed on both polyclonal and monoclonal level in humans [11, 12], as well as in the sera of mice immunized with a protein bearing a single type of PTM [13]. Together, this indicates that the different anti-modified protein antibodies (AMPA)—ACPA, anti-CarP, and AAPA—may evolve from a common precursor.

In most RA patients, AMPA response is dominated by ACPA as the most prevalent reactivity, potentially suggesting a possible role for anti-CarP and AAPA as epiphenomena of ACPA. However, it is unknown whether ACPA are also predominant prior to the disease onset, as the data regarding AMPA reactivities in pre-RA individuals are scarce: AAPA are a very recent discovery, and their prevalence before the onset of RA (and in relationship to ACPA) has thus far been investigated in a very limited number of cohorts [14]. Anti-CarP (while often being co-present with ACPA) have been shown to be positive in ACPA-negative individuals in two independent pre-disease cohorts [15, 16].

Due to the multireactive nature of the AMPA response and the relationship between ACPA epitope spreading and disease onset, it is important to understand the evolution of the PTM-recognition profile to enhance the comprehension of RA pathophysiology. We therefore set out to investigate (1) how the PTM-recognition profile changes over time in individuals at risk of RA and in patients within the first year of disease and (2) how controlled subsequent exposure to different PTMs shapes the AMPA response in mice.

## Materials and methods

### Protein modifications

All procedures for protein modification and immunizations were performed as described previously [13]. In short, carbamylation was achieved by incubating the proteins, namely ovalbumin and fibrinogen (starting concentration between 1 and 5 mg/ml), with 0.5 or 1 M potassium cyanate, after which they were extensively dialysed in phosphate buffered saline (PBS) for 3 days. Acetylation was achieved by diluting proteins (starting concentration 1 mg/ml) in 0.1 M Na_2_CO_3_, then treating with acetic anhydride and subsequently with pyridine. Proteins were incubated at 30 °C for 5 h or overnight while shaking. After incubation, the acetylation reaction was stopped by adding 1 M Tris. Acetylated proteins were purified by exchanging the buffer for PBS through Zebra Spin Desalting columns (Thermo Scientific). Citrullination of fibrinogen was performed by incubation of the protein (starting concentration 1 mg/ml) with Peptidyl Arginine Deiminase (PAD) 4 enzyme (Cat# 1584, Sigma Aldrich) in the presence of 0.1 M Tris-HCl (pH 7.6) and 0.15 M CaCl_2_. For fibrinogen, 5 units of PAD were added per mg of protein and then incubated overnight at 53 °C. Modification of fibrinogen and OVA were validated by ELISAs using commercial polyclonal rabbit anti-carbamyllysine (anti-homocitrulline) antibodies (Cat# STA-078, Cell Biolabs), commercial polyclonal rabbit anti-acetyllysine antibodies (Cat# ADI-KAP-TF120-E, Enzo Lifesciences), a patient-derived monoclonal ACPA (described previously [12]), and controlled for impurities by mass spectrometry as described previously [13].

### AMPA-positive individuals

Human serum samples for this study included two groups of donors, having participated in previously published studies: individuals at risk of RA from the Nagasaki Island Study [17] and RA patients from the IMPROVED study [18].

The sample pre-selection was based on (1) the availability of longitudinal samples (for IMPROVED, from all four timepoints) and (2) AMPA positivity according to the pre-existing serological data from the original studies (for > 1 AMPA type in case of IMPROVED). In case of the Nagasaki Island Study, the pre-existing data included only ACPA-positivity (as measured by CCP2 ELISA, Supplementary Table 1). The samples were collected at two timepoints with three years in between. At the first timepoint, all eight pre-selected individuals did not have RA diagnosis; at the second timepoint, one individual was diagnosed with RA (at-risk individual #7). Another at-risk individual (#8) developed RA after the second timepoint.

For the IMPROVED study, the pre-existing data included positivity for ACPA (as measured by CCP2 ELISA, Supplementary Table 1), anti-CarP (carbamylated fetal calf serum ELISA), and AAPA (acetylated vimentin peptide ELISA, Orgentec); positivity for at least two of these reactivities at one timepoint was required for inclusion. The IMPROVED study initially included 399 patients with early (< 2 years) untreated RA, who were actively treated during the study [19]. Serum was collected at baseline and after 4, 8, and 12 months (Supplementary Tables 2 and 3). The IMPROVED patients were previously shown to have an AMPA decrease [7], likely being the effect of treatment (mostly methotrexate [20]), which was not the primary interest of the current study. To tackle that, an extra pre-selection criterion was introduced, namely a sero-conversion of at least one AMPA reactivity occurring between the observation timepoints. This extra criterion, however, limited the amount of eligible patients to 10. All patients with RA fulfilled the European League Against Rheumatism/American College of Rheumatology 2010 classification criteria. The protocols of both studies were approved by the local ethics committees and all patients provided written informed consent.

### Mouse immunizations

Seven- to 8-week-old female C57BL6/J mice were purchased from Charles River Laboratories. Two immunization experiments with similar design were performed: 1st experiment included 6 mice per immunization group; 2nd experiment included 7 mice per group (Supplementary Table 4). Mice received two injections intraperitoneally with antigen (PTM Ovalbumin) (100 μg) emulsified 1:1 in Alhydrogel (Cat# vac-alu-250, Invivogen). The period between the first and the booster immunization was 5 weeks. Animal experiments were approved by the Ethical Committee for Animal Experimentation of the LUMC, Leiden. All immunized mice were healthy and showed no signs of arthritis throughout the experiment. We did not immunize mice with citrullinated ovalbumin, as it failed to induce a robust AMPA response [13].

### Detection of anti-modified-protein antibodies

For the detection of AMPAs in mice, the following Enzyme-Linked ImmunoSorbent Assay (ELISA) was performed: modified proteins (i.e., modified fibrinogen, ModFib) or their non-modified counterparts were coated at a concentration of 10 μg/mL in 0.1 M carbonate-bicarbonate buffer (pH 9.6) overnight on Corning polystyrene 384-well microplates. The plates were blocked with PBS + 1% BSA. The mouse sera were diluted in RIA buffer (10 mM TRIS-HCl (pH 7.6), 350 mM NaCl, 1% Triton-X100, 0.5% sodium deoxycholate, 0.1% SDS) and incubated overnight. Mouse serum was titrated starting from 1:25 to 1:51200 with 1:2 steps. Human samples were titrated from 1:50 to 1:51200 with 1:2 steps. All samples of the same individual were measured on the same plate. Experiments were done twice, unless available mouse serum was limited due to blood collection from the living animals. When multiple plates were used, two reference samples were included on every plate. Samples were analyzed in single wells due to the limited volumes being available. Binding of mouse IgG was detected with horseradish Peroxidase (HRP)-conjugated goat-anti-mouse IgG1 (Southern Biotech) and subsequently visualized with ABTS (Sigma Aldrich). Washing steps were performed between each incubation with PBS + 0.05% Tween-20 (Sigma). All incubations, aside from the incubations with goat-anti-mouse IgG1 and ABTS, were performed at 4 °C, while the final two steps were performed at room temperature (RT). For the detection of AMPA in human sera, a similar ELISA was used for testing reactivity against ModFib, with HRP-conjugated rabbit anti-human IgG (DAKO) used for detection. ELISA with coated cyclic modified peptide 4 (CModP4) variants was performed as described previously [12].

### Use of antibody titers to compare different responses

The term “titer” has traditionally been used to describe the highest dilution of a serially diluted sample at which the reaction (agglutination, precipitation, lysis, etc.) is still visible [21]. Titer (defined as the greatest serum dilution still giving a positive signal) is judged a more robust readout for comparison of antibody levels as compared to arbitrary units. Measuring arbitrary units at one dilution does not account for differences between titration curves of the standard and individual samples, making it less reliable than measuring titer [22]. In our ELISA experiments, we determined the titer as the highest dilution at which the optical density (OD) value reaches the pre-determined cut-off of an OD = 0.1.

As described previously [13], we used a different backbone (fibrinogen instead of ovalbumin) for ELISA readouts of the murine experiments to be able to discriminate the reactivity against PTMs from the reactivity against the (Ovalbumin) backbone used for immunization. All murine serum samples were serially diluted; delta-OD values were calculated by subtracting an average blank OD value from the sample measurements. Curve fitting was applied for delta-OD values of the titrated sample in GraphPad Prism v8, and the dilution factor for the given OD (0.1) was selected as titer (Supplementary Fig. 1). If the lowest delta-OD value was above OD of 0.1, the dilution factor was extrapolated using the fitted curve. The titers of anti-CarP and AAPA responses were then used to compare the relative abundance of antibodies recognizing antigens with respective PTMs. As the antibody-titer data have exponential nature, geometric means were used.

### Statistics

Statistical tests were performed with Prism V.8 (GraphPad). Differences in titer were tested with Mann-Whitney *U* tests. A *p* value of < 0.05 was considered significant.

## Results

### Dynamic changes of AMPA-reactivity titers in humans

**Fig. 1:**
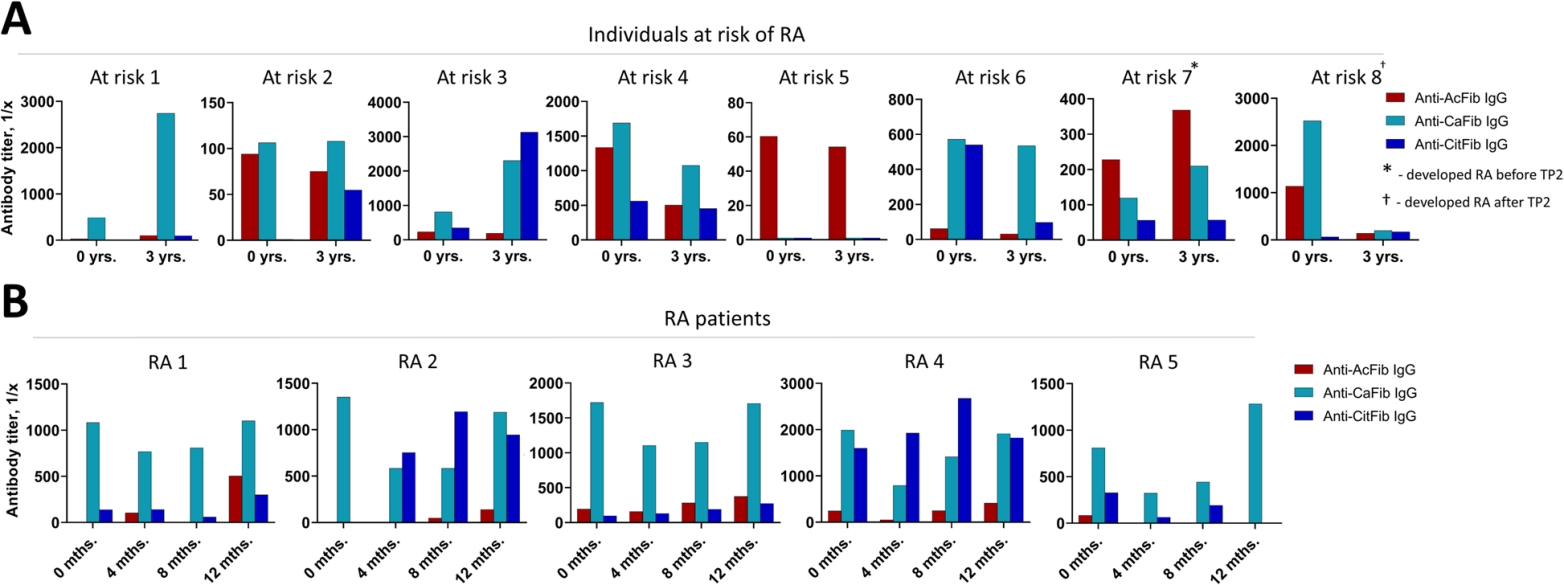
The shift of the AMPA reactivity in individuals at risk of RA and RA patients. **A**, **B** Different AMPA (targeting citrullinated, carbamylated or acetylated antibodies) titers as calculated by ELISA with modified fibrinogen. Human serum samples collected at different timepoints were measured. The data are depicted as clustered columns to simplify comparison of each AMPA reactivity between the timepoints, different PTM-reactivities should not be compared directly. The difference between the timepoints was 3 years for individuals at risk and 4 months for RA patients. Shown are inverted AMPA titers of 8 individuals at risk of RA and 5 RA patients

Previously, it has been shown that AMPA levels can fluctuate over time [20, 23]; however, these levels were measured in arbitrary units at a fixed dilution, and different AMPA reactivities were determined using different antigen backbones. To determine whether the quantitative fluctuations of different AMPA reactivities can also be observed by analyzing titers and by using the same antigen backbone, we measured AMPA titers in longitudinally collected serum of individuals at risk of RA and RA patients. First, we used the three ModFib ELISAs with fixed dilutions (1:50) to screen for AMPA (ACPA, AAPA, anti-CarP) positivity in serum samples from the pre-selected individuals at risk of RA (*n* = 8) and RA patients (*n* = 10) (data not shown). Positivity for at least two types of AMPA was found in 8/8 samples of the individuals at risk of RA and in 5/10 RA patients (see clinical characteristics of the RA patients in Supplementary Table 5). To investigate temporal changes of the AMPA reactivities, these samples were then titrated and measured on the ModFib ELISAs to determine AMPA titers. The different AMPA reactivities were found to follow different trends relative to each other, while overall response tendencies appeared to be unique in every individual (Fig. 1). For example, the ACPA-response, as measured by antibody titer to citrullinated fibrinogen increased considerably in “at-risk individual 2,” whereas anti-Carp-antibody and AAPA titers remained, by and large, unchanged. A similar increase in ACPA-response was observed in “at-risk individual 3,” whereas, for example, in “at-risk individual 6,” a decrease in ACPA titers was observed in time (Fig. 1A). Thus, although no consistent changes were observed in absolute or relative (to each other) specific AMPA-levels in individuals at risk for RA, these data show that the AMPA-response to a given PTM is not stable and that these can fluctuate in time. Similar data were obtained in RA (Fig. 1B), indicating that also in established disease, the (relative) levels to different PTM can fluctuate in time. When these samples were tested on ELISA coated with different variants of CModP4 peptides, we observed titer fluctuations between different timepoints; however, these changes only partially overlapped with the results of the ModFib ELISA, likely due to the different antigenic backbone (data not shown).

### Induction of cross-reactive anti-PTM memory in mice

To understand the immunological mechanism behind this observation, we next performed immunization studies in mice thereby controlling the timing of exposure to different and defined PTMs. Building on our previous observations showing the induction of robust AMPA responses [13], we immunized mice with acetylated or carbamylated ovalbumin (AcOVA, CaOVA). To investigate whether cross-reactive AMPA responses could be boosted and to which extent these responses can be diverted towards another PTM-reactivity, mice were either immunized twice with a protein bearing one type of PTMs (solely acetylated or solely carbamylated ovalbumin, AcOVA or CaOVA) or consecutively with antigens bearing different PTMs (first AcOVA, then CaOVA or vice versa).

**Fig. 2:**
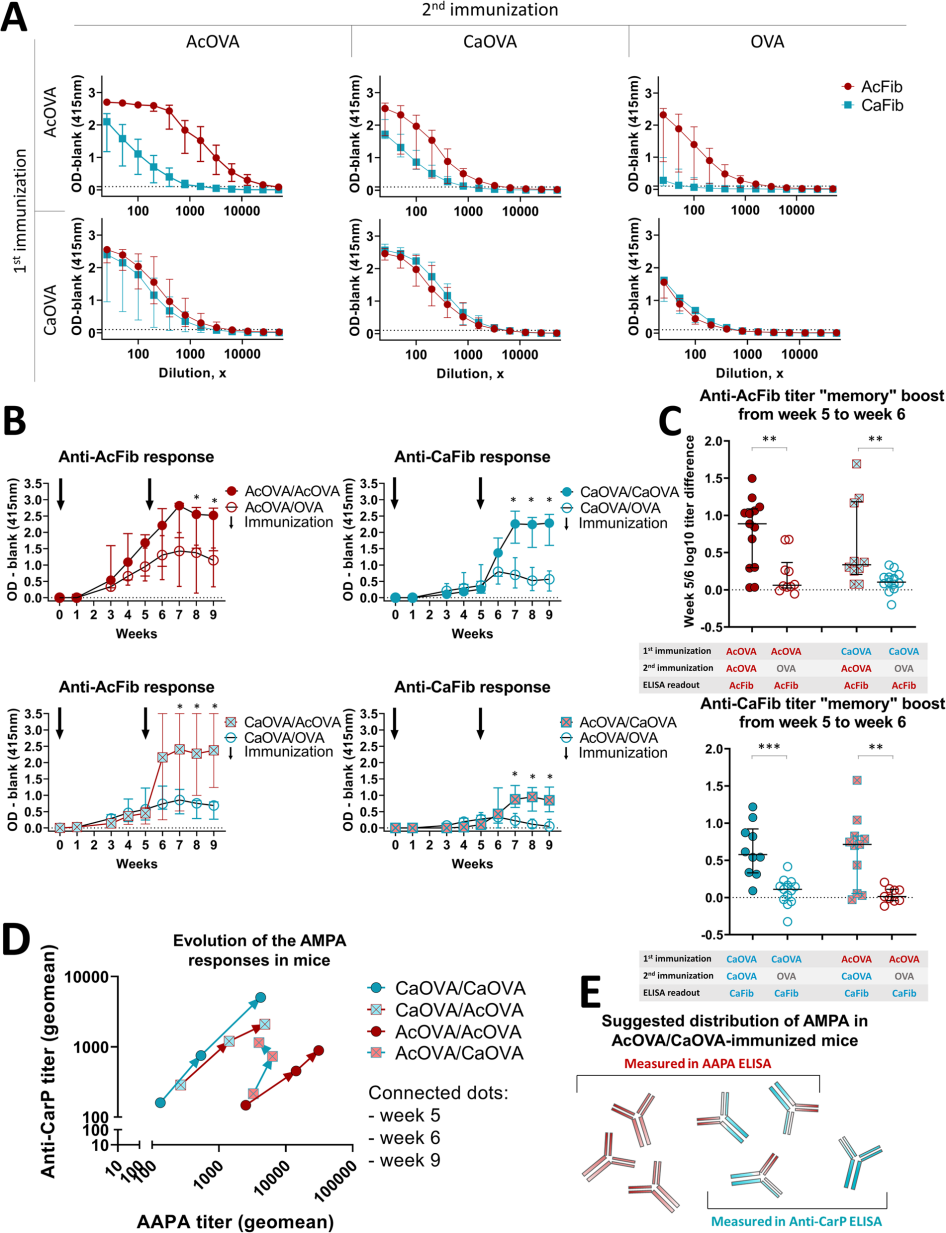
Evolution of the AMPA-reactivity profile in mice immunized with different PTM-antigens. **A** ELISA of the titrated murine sera showing the development of anti-modified protein antibody (AMPA) response to different post-translational modifications of fibrinogen, namely acetylated (AcFib) and carbamylated (CaFib) variants. Individual graphs depict titration ELISA results measured in the end of the experiment in mice groups with different immunization strategies. Medians and interquartile intervals per serum dilution are shown. Immunization experiments were performed twice with similar results. Data from one of the two representative immunization experiments are shown. **B** Timelines demonstrating median anti-AcFib and anti-CaFib reactivity of the immunized mice per immunization group. Arrows indicate the timepoints at which the mice were immunized (week 0 and week 5). Graphs depict ELISA OD-s measured with sera collected at different timepoints and diluted 1:50 for ELISA; medians and interquartile intervals are shown per timepoint. Data from one of the two representative immunization experiments are shown. *p* values (asterisk) refer to the change between two immunization groups within one timepoint according to Mann-Whitney *U* test (*p < 0.05). **C** Changes in AMPA titers of individual mice within 1 week after the booster (between timepoints 5 and 6), calculated by subtraction of the log-transformed timepoint 5 titer from the timepoint 6 titer; means ± SD are shown per immunization group. Pooled titer data from two immunization experiments are shown. *p* values (asterisk) refer to the change between two immunization groups according to Mann-Whitney *U* test (*p < 0.05, **p < 0.01, ***p < 0.001). All experiments were performed twice with similar results. The data obtained from one experiment is shown. **D** Relative evolution of the anti-CarP and AAPA responses: the titer evolution data were summarized per immunization group by depicting average titers. Due to the exponential nature of titer values, geometric mean was used to determine the average. Pooled titer data from the two immunization experiments are shown, measured with serum collected at week 5, week 6, and week 9. Example of performed calculations is shown in supplementary methods. **E** Model illustrating multireactive nature of AMPA is shown together with which antibodies are measured in AMPA assays applied in the study

As shown in Fig. 2A, immunization with either PTM-antigen led to a cross-reactive AMPA response, although the degree of cross-reactivity differed between the two investigated PTMs. Mice immunized twice with acetylated ovalbumin (AcOVA) developed an high-titer antibody response against acetylated antigens that displayed a limited reactivity against carbamylated antigens as at least 10 times lower titer to carbamylated fibrinogen was noted when measured at the end-of-experiment timepoint (Fig. 2D). However, mice immunized twice with carbamylated ovalbumin (CaOVA) developed a highly cross-reactive AMPA response, as similar antibody-titers were measured towards both carbamylated and acetylated antigens. Robust ACPA response (measured with citrullinated fibrinogen) could not be detected in these mice (Supplementary Fig. 2), which was similar to what we observed previously [13].

### Immunization with an antigen bearing one type of PTM induces memory that can be boosted by another PTM

Subsequently, we compared AMPA-titer changes in immunized mice boosted with the “opposing” PTM. Irrespective of the PTM used for the first immunization, a booster immunization with the other PTM resulted in increased reactivity to the second/booster PTM, indicating that immunization with a defined PTM-antigen leads to the generation of anti-PTM memory B cells able to cross-recognize antigens with other PTMs (Fig. 2B, Supplementary Fig. 3). This is further illustrated by a comparison of AAPA and anti-CarP titers after booster immunization, revealing a titer increase in most cross-immunized mice within one week after the second immunization (Fig. 2C). Importantly, when the booster immunization antigen did not contain PTMs as control (AcOVA/OVA and CaOVA/OVA mice), the AMPA response did not expand or increase, but rather demonstrated a trend for decline over time (Fig. 2B). Together, these data indicate that immunization with a defined PTM-antigen leads to the generation of anti-PTM memory B cells able to cross-recognize antigens with other PTMs and that can be (re)activated by different PTMs.

### Booster immunization with a different PTM skews AMPA-reactivity profile

The presented data also indicate that secondary exposure (involving a PTM, different from the PTM initiating the response) might affect the reactivity of the AMPA response as a whole. To obtain more insight into this possibility, we quantified AAPA and anti-CarP-antibody-titer changes when mice are exposed to different PTMs in time. Temporal relationship between AMPA titers recognizing carbamylated and acetylated residues was investigated by comparing geometrical means of anti-AcFib and anti-CaFib titers between different immunization groups. As depicted in Fig. 2D, immunization with AcOVA followed by boosting with CaOVA skewed the AMPA-response towards higher antibody-titers against carbamylated antigens. A similar relative stronger increase in antibody-titer to the “booster PTM” was observed in mice first immunized carbamylated antigen, followed by exposure to acetylated protein. Together, these data indicate that the overall AMPA-response profile can shift towards a “booster” PTM as shown by (relative) increases in AMPA-titer to a particular PTM in time.

## Discussion

Our previous work in mice revealed that immunization with an antigen harboring one PTM can give rise to a broad immune response recognizing multiple PTMs [13]. It was, however, not known how consecutive exposure to different PTMs affects the AMPA reactivity profile. In the current study, we extend these observations with data indicating that an AMPA response initiated by one PTM cannot only be boosted by the same, but also by other PTMs, indicating that memory B cells are not “fixed” in their PTM-recognition profile. Likewise, we observed that the overall AMPA-reactivity profile can change over time.

Immunization with CaOVA as well as with AcOVA induces a cross-reactive AMPA response; however, the degree of cross-reactivity appears to be substantially higher in case of CaOVA immunization. It is unknown what causes this difference, but these observations may point to differences in the B cell receptor repertoires responding to these PTMs. Apparently, BCRs responding CaOVA can also recognize acetyllysine whereas not all BCRs responding to acetyllysine also react to homocitrulline. Consecutive immunization with different PTM-antigens thus leads to a more balanced distribution (Fig. 2D, E).

The AMPA response is a hallmark of RA, but its development and evolution is still poorly understood. ACPA, anti-CarP, and AAPA appear to be closely related, yet a large variation in modified antigen-recognition profile among individuals is observed [12]. Our study suggests a potential explanation for these individual differences providing a mechanism explaining how consecutive exposure to various PTMs shapes AMPA-response evolution.

Our data suggest that consecutive immunizations of mice with two different PTMs lead to a rapid boost in the response targeting the second immunizing PTM. This response pattern can be explained in two ways that do not exclude each other: a de novo response involving priming of naïve B cells and/or boosting of the memory cells originating from the primary antigen-exposure. Although newly recruited naïve B cells likely play a role in the secondary response, they emerge only gradually [24]. In our study, we observed a booster effect already after 1 week (Fig. 2B, C), suggesting a role for the existing memory B cells.

Once established, the AMPA response is likely to be triggered every time the immune system encounters a PTM-antigen. This ongoing antigenic triggering is also indicated by previous observations showing that ACPA-expressing B cells exhibit an activated phenotype at different stages of RA [25]. Likewise, given the half-life of IgM, the continued presence of ACPA IgM at different stages of arthritis also points to continuous activation of the AMPA response [26]. We now show that subsequent exposure to antigens harboring other PTMs can skew the overall AMPA-recognition profile towards the more predominant recognition of later encountered PTMs. Evidence for skewing is not only coming from our mouse studies, but can also be taken from the observations that the titers of different AMPA subsets can fluctuate in time. In fact, these fluctuations may be even larger than what is evident from the serum antibody measurements, as there is likely a basal level of AMPA production maintained by long-lived plasma cells producing AMPA with a “fixed” PTM-recognition profile [27].

Thus, the PTM involved in initial response induction and the PTM predominantly recognized in, e.g., established disease may differ. Despite the previous observations showing that ACPA dominate the AMPA response in RA patients [18, 28], it is unknown whether ACPA also dominate the AMPA response prior to disease onset. Based on the limited human data of this study alone, it is a challenge to conjure a clear picture of AMPA reactivity transitions in RA either before or after the disease onset. However, the mouse data of this study do provide a mechanism which could underlie a transition from one dominant AMPA reactivity to another. Importantly, it has also been proving challenging to identify one citrullinated culprit antigen involved in the breach of tolerance towards PTMs [29]. Additionally, different PTM-antigens triggering the immune system can also originate from different locations in the body. Thus, based on our data, it could be merely speculated that an AMPA response could be initiated by acetylated (or carbamylated) foreign antigens present at mucosal surfaces [30] and further expanded and skewed by citrullinated proteins, present in synovial joints [31, 32].

The de novo generation of PTM-antigens at different sites is likely to be driven by inflammation. One of the most frequently proposed mechanisms suggests the role for neutrophil death accompanied with the formation of so-called neutrophil extracellular traps (NETs) which was suggested to be linked with citrullination [33, 34], acetylation [35], and carbamylation [36]. In our mouse work, injection of a protein together with an adjuvant likely induced a local inflammatory process (not formally assessed during the study). However, immunizing a mice with a non-PTM antigen did not lead to AMPA production, suggesting that an inflammatory process needs to meet certain requirements in order to generate PTM-antigens capable of inducing an AMPA immune response.

An important pitfall complicating the interpretation of our mouse data is related to the fact that we are modeling AMPA responses in mice by looking predominantly at anti-CarP and AAPA, while in RA patients, the AMPA response is mainly dominated by ACPA. This is mainly due to the difficulties in inducing ACPA in mice. On the one hand, when we used a citrullinated antigen for mouse immunization (such as citrullinated ovalbumin), we did not observe AMPA development. Moreover, in our previous [13] and current work, we could observe antibodies recognizing acetylated and carbamylated versions of fibrinogen, but not citrullinated fibrinogen. The absence of ACPA development upon immunization with CitOVA could potentially be explained by reduced efficiency of enzymatic citrullination as compared to chemically induced acetylation or carbamylation. However, the absence of ACPA upon immunization with acetylated or carbamylated ovalbumin could be explained by different reasons: (1) potential importance of backbone surrounding the PTM that could differ for lysine (modified to become acetyllysine or homocitrulline) and arginine (modified to become citrulline), thus increasing the likelihood of cross-reactivity between responses targeting modifications of the same amino-acid (lysine); (2) another potential reason could be that the immune system of mice may for unknown reasons be more tolerant towards citrullinated antigens as compared to carbamylated or acetylated antigens. Although challenging, there could be various ways to overcome this pitfall: on the one hand, immunization and detection strategy could be expanded by immunizing mice with a mixture of PTM-proteins and using a range of other, unrelated PTM-proteins to detect the AMPA reactivities. On the other hand, mice of other strains and other animals, such as rabbits could be used for similar immunization experiments. Despite these caveats, we believe that the relationships within the AMPA response observed in mice, are nevertheless insightful regarding the nature of cross-reactive anti-PTM responses and thus relevant for the AMPA response observed in RA patients.

The current study is the first to demonstrate a potential mechanism explaining the temporal changes in the PTM-recognition profile. However, our study has several limitations: first, the human arm of the study and its impact are rather limited, as these data mainly confirm the previous observations indicating that AMPA levels fluctuate over time [20, 23]. Importantly, however, these results were obtained with a potentially more accurate method (Supplementary Fig. 4). A second limitation is that we analyzed AMPA reactivities by primarily using modified human fibrinogen for ELISA readout. It may well be that using other backbones for analysis could result in different dynamics in AMPA reactivities, especially in humans. This would not, however, alter the interpretation, as it would only suggest that the existing AMPA response can be triggered by PTM-antigens with different backbones and the backbone itself plays a role in the skewing of the AMPA reactivity profile.

## Conclusion

Overall, these data illustrate the dynamic relation between different reactivities within the AMPA response. The nature of the initial PTM-antigen determines the degree of cross-reactivity, but skewing of the overall reactivity pattern can be triggered by subsequent exposure to other PTMs. These data might explain temporal differences in the AMPA-response profile and point to the possibility that the PTM responsible for the initiation of the AMPA response may differ from the PTM predominantly recognized later in time.

## Supplementary Information

**Additional file 1: Supplementary Methods**. Calculation of summary titer evolution (as implemented for Fig. 2D). **Supplementary Table 1**. CCP2 ELISA measurements prior to the study. **Supplementary Table 2**. **Supplementary Table 3**. Changes in AMPA status in the IMPROVED cohort. **Supplementary Table 4**. Overview of the mice immunization groups. **Supplementary Table 5**. Detailed information on RA patients included in the study. **Supplementary Fig. 1**. AMPA ELISA titration curve examples. **Supplementary Fig. 2**. Citrullinated and unmodified fibrinogen ELISA with sera from mice immunized with modified ovalbumin. **Supplementary Fig. 3**. Timeline showing evolution of AMPA reactivities in mice with differing first-immunization antigens. **Supplementary Fig. 4**. Example comparing level vs titer analyses of anti-AcFib and anti-CaFib reactivities in AcOVA/AcOVA-immunized mice.

## Data Availability

The datasets used and/or analyzed during the current study are available from the corresponding author upon reasonable request.

## Abbreviations

RA: Rheumatoid arthritis
ACPA: Anti-citrullinated protein antibodies
PTM: Post-translational modification
AAPA: Anti-acetylated protein antibodies
Anti-CarP: Anti-carbamylated protein antibodies
anti-MAA: Anti-malondialdehyde-acetaldehyde antibodies
AMPA: Anti-modified protein antibodies
PBS: Phosphate buffered saline
AcOVA: Acetylated ovalbumin
CaOVA: Carbamylated ovalbumin
OD: Optical density
AcFib: Acetylated human fibrinogen
CaFib: Carbamylated human fibrinogen
DMARDs: Disease modifying anti-rheumatic drugs
