# Supplementary material for: Evolution of anti-modified protein antibody responses can be driven by consecutive exposure to different post-translational modifications
Source: Arthritis Res Ther. 2021 Dec 8;23:298. doi: 10.1186/s13075-021-02687-5 (PMC8653599; doi:10.1186/s13075-021-02687-5)
Supplement: Supplementary file 1 — Additional file 1: Supplementary Methods. Calculation of summary titer evolution (as implemented for Fig. 2D). Supplementary Table 1. CCP2 ELISA measurements prior to the study. Supplementary Table 2. Supplementary Table 3. Changes in AMPA status in the IMPROVED cohort. Supplementary Table 4. Overview of the mice immunization groups. Supplementary Table 5. Detailed information on RA patients included in the study. Supplementary Fig. 1. AMPA ELISA titration curve examples. Supplementary Fig. 2. Citrullinated and unmodified fibrinogen ELISA with sera from mice immunized with modified ovalbumin. Supplementary Fig. 3. Timeline showing evolution of AMPA reactivities in mice with differing first-immunization antigens. Supplementary Fig. 4. Example comparing level vs titer analyses of anti-AcFib and anti-CaFib reactivities in AcOVA/AcOVA-immunized mice. [file 13075_2021_2687_MOESM1_ESM.docx]

**Supplementary material**

**Supplementary methods. calculation of summary titer evolution (as implemented for Figure 2D)**

Dilution factors are log10 transformed and standard curves are interpolated using Graphpad Prism (Asymmetric sigmoidal 5PL), dilution for 0.1 OD is determined (see figure).

Log10 dilution at 0.1 OD is then transformed back (10^[Log10 factor]). Summary titer is calculated as geometric mean of the dilution factor values per reactivity (AAPA or anti-CarP) per immunization group (see table).

**AcFib ELISA**

**Experiment 1**

**AcFib ELISA**

**Experiment 2**

Acetylated fibrinogen ELISA with the titrated serum of the mice immunized twice with acetylated ovalbumin. End-of-experiment (week 9) data of the two immunization experiments are shown, each curve represents one mouse.

| **Log10 dilution factor (titer)** |  | **Dilution factor (titer)** |  | **Geomean titer** |
| --- | --- | --- | --- | --- |
| 5.14 |  | 137503 |  | 44598 |
| 5.25 |  | 176166 |  |  |
| 4.39 |  | 24399 |  |  |
| 4.50 |  | 31439 |  |  |
| 5.39 |  | 245370 |  |  |
| 4.33 |  | 21524 |  |  |
| 4.60 |  | 40212 |  |  |
| 5.02 |  | 105721 |  |  |
| 4.64 |  | 43970 |  |  |
| 4.31 |  | 20378 |  |  |
| 4.41 |  | 26139 |  |  |
| 3.46 |  | 2851 |  |  |
| 5.00 |  | 99108 |  |  |

Summary of the calculations to determine geometric mean titer.

**Supplementary table 1. CCP2 ELISA measurements prior to the study**

| Individuals at risk of RA | | | | | | | | | | | | |
| --- | --- | --- | --- | --- | --- | --- | --- | --- | --- | --- | --- | --- |
| CCP2 AU/ml | At-risk #1 | At-risk #2 | | At-risk #3 | At-risk #4 | | At-risk #5 | | At-risk #6 | At-risk #7 | | At-risk #8 |
| 0 years | 64 | 72 | | 387 | 807 | | 29 | | 361 | 36 | | 171 |
| 3 years | 361 | 197 | | 476 | 1160 | | 33 | | 206 | 56 | | 111 |
| RA patients | | | | | | | | | | | | |
| CCP2 AU/ml | RA #1 | | RA #2 | | | RA #3 | | RA #4 | | | RA #5 | |
| 0 months | 236 | | 41 | | | 1600 (max) | | 91 | | | 496 | |
| 4 months | 328 | | 262 | | | 1600 (max) | | 478 | | | 555 | |
| 8 months | 395 | | 368 | | | 1600 (max) | | 547 | | | 726 | |
| 12 months | 1519 | | 1009 | | | 1600 (max) | | 960 | | | 1137 | |

**Supplementary table 2**

| **AMPA status** | **0 mths (n=356)** | **4 mths (n=225)** | **8 mths (n=209)** | **12 mths (n=212)** |
| --- | --- | --- | --- | --- |
| ACPA-/CarP-/AAPA- | 46 | 41 | 35 | 32 |
| ACPA-/CarP-/AAPA+ | 3 | 1 | 1 | 0 |
| ACPA-/CarP+/AAPA- | 14 | 6 | 5 | 1 |
| ACPA-/CarP+/AAPA+ | 1 | 0 | 0 | 0 |
| ACPA+/CarP-/AAPA- | 87 | 93 | 75 | 73 |
| ACPA+/CarP-/AAPA+ | 45 | 42 | 34 | 51 |
| ACPA+/CarP+/AAPA- | 79 | 22 | 27 | 32 |
| ACPA+/CarP+/AAPA+ | 81 | 20 | 32 | 23 |

Overview of the AMPA status at different timepoints, as measured in serum of the RA patients included in the IMPROVED.

**Supplementary table 3. Changes in AMPA status in the IMPROVED cohort.**

| **AMPA status change** | **ACPA (anti-CCP2) IgG** | **Anti-CarP (anti-CaFCS) IgG** | **AAPA (anti-acetyllysine vimentin peptide) IgG** |
| --- | --- | --- | --- |
| Stable positive | 163 | 46 | 59 |
| Pos to neg | 4 | 47 | 19 |
| Neg to pos | 5 | 9 | 18 |

Overview of the AMPA status changes in 252 seropositive patients of the IMPROVED study that had AMPA data available for at least two timepoints.

**Supplementary table 4. Overview of the mice immunization groups**

| Mice immunization groups | Experiment 1 | Experiment 2 |
| --- | --- | --- |
| AcOVA/AcOVA | 6 | 7 |
| AcOVA/CaOVA | 6* | 7 |
| AcOVA/OVA | 6 | 7 |
| CaOVA/CaOVA | 6* | 7 |
| CaOVA/AcOVA | 6ᶧ | 7 |
| CaOVA/OVA | 6 | 7 |

Numbers of mice included per immunization group in each of the two immunization experiments. For several mice, there was not enough blood available to perform titration ELISA for timepoint 6, due to limitations when collecting blood from the living mice. These mice were not included in the analysis presented on figures 2C and 2D.

*: two mice were excluded due to lack of material at timepoint 6.

ᶧ: three mice were excluded due to lack of material at timepoint 6.

**Supplementary table 5. Detailed information on RA patients included in the study**

|  | Age | Gender | RA diagnosis according to ACR/EULAR 2010 | Symptom duration | DAS at baseline | Remission at timepoint 2 |
| --- | --- | --- | --- | --- | --- | --- |
| RA #1 | 56 | F | + | 52 weeks | 3.60 | No |
| RA #2 | 76 | M | + | 7 weeks | 4.51 | No |
| RA #3 | 27 | M | + | 16 weeks | 3.48 | Yes |
| RA #4 | 65 | F | + | 35 weeks | 3.42 | No |
| RA #5 | 39 | M | + | 27 weeks | 2.50 | Yes |

Clinical characteristics of the IMPROVED patients, of who AMPA titers were analyzed

**At-risk #3 titers**

**B**

**At-risk #3, 0 years**

**At-risk #3, 3 years**

**A**


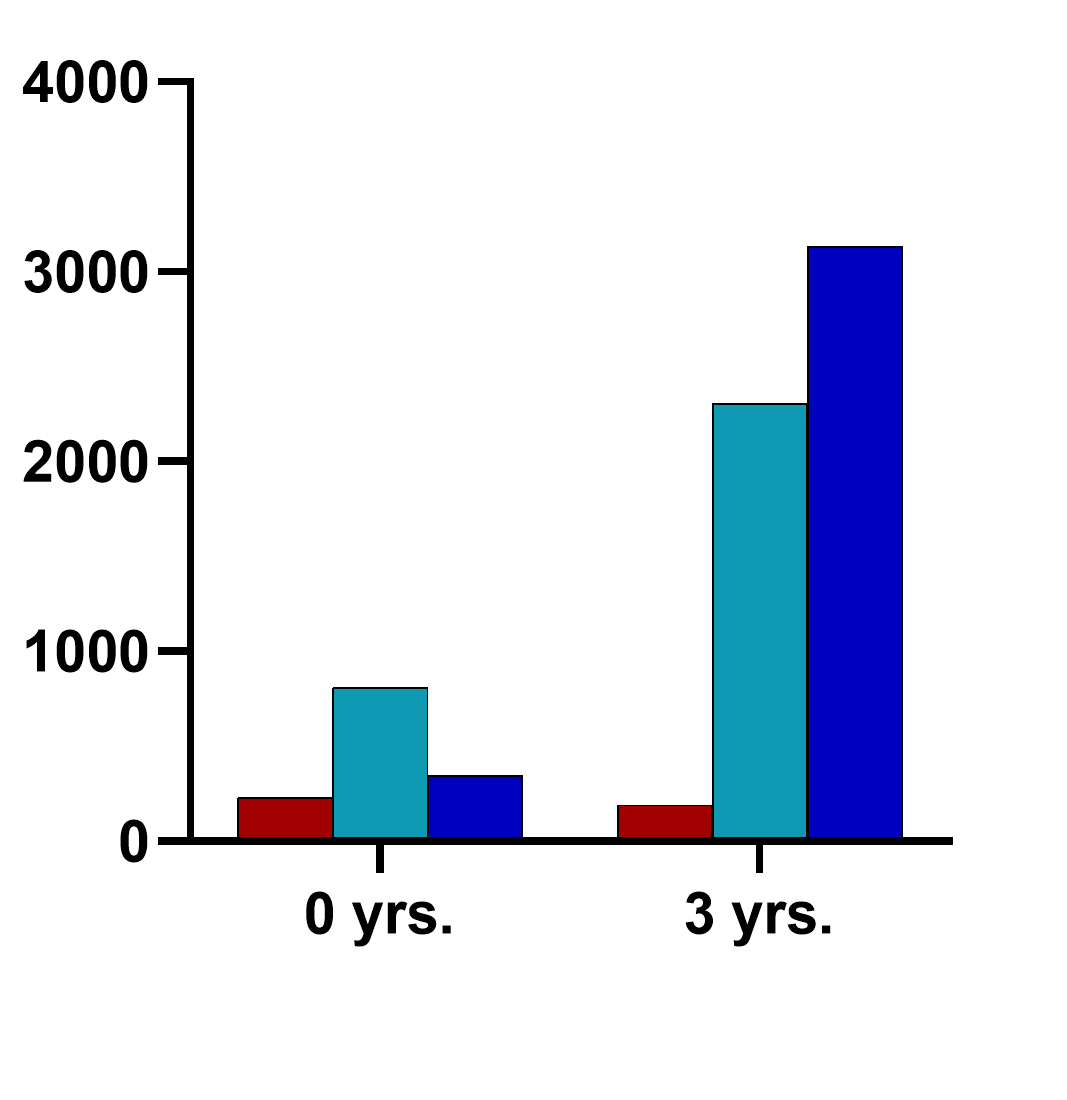


**Titer, 1/x**

**Supplementary Figure 1. AMPA ELISA titration curve examples.**

Examples of titration curves measured on modified fibrinogen ELISA (A) and the resulting bar graphs (B) of two timepoints in one at-risk individual.

**B**

**A**

**Supplementary figure 2. Citrullinated and unmodified fibrinogen ELISA with sera from mice immunized with modified ovalbumin**

A-B: ELISA of 1:50 diluted mice serum collected at the end-of-experiment (week 9) timepoint. Data of one immunization experiment are shown, n = 6 mice per immunization group. Data are depicted per immunization group, each dot represents one mouse.

**B**

**A**


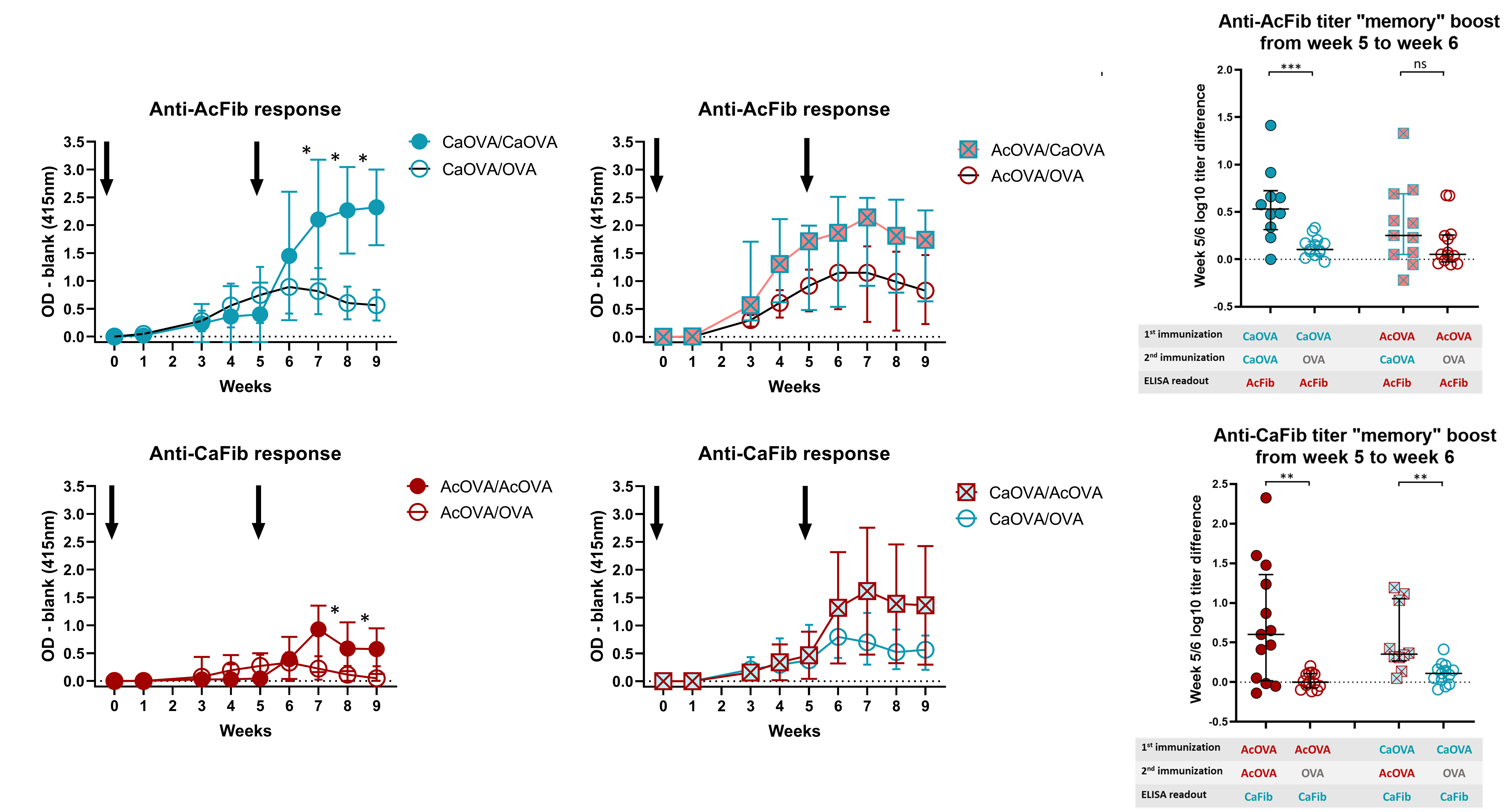


**Supplementary figure 3. Timeline showing evolution of AMPA reactivities in mice with differing first-immunization antigens**

A: Timelines demonstrating median anti-AcFib and anti-CaFib reactivity of the immunized mice per immunization group. Arrows indicate the timepoints at which the mice were immunized (week 0 and week 5). Graphs depict ELISA OD-s measured with 1:50 diluted sera collected at different timepoints; medians and interquartile intervals are shown per timepoint. Data from one of the two representative immunization experiments are shown. p values (asterisk) refer to the change between two immunization groups within one timepoint according to Mann-Whitney U test (* p < 0.05).

B: Changes in AMPA titers of individual mice within one week after the booster (between timepoints 5 and 6), calculated by subtraction of the log-transformed timepoint 5 titer from the timepoint 6 titer, means ± SD are shown per immunization group. Pooled titer data from two immunization experiments are shown. p values (asterisk) refer to the change between two immunization groups according to Mann-Whitney U test (* p < 0.05, **p < 0.01, ***p < 0.001).


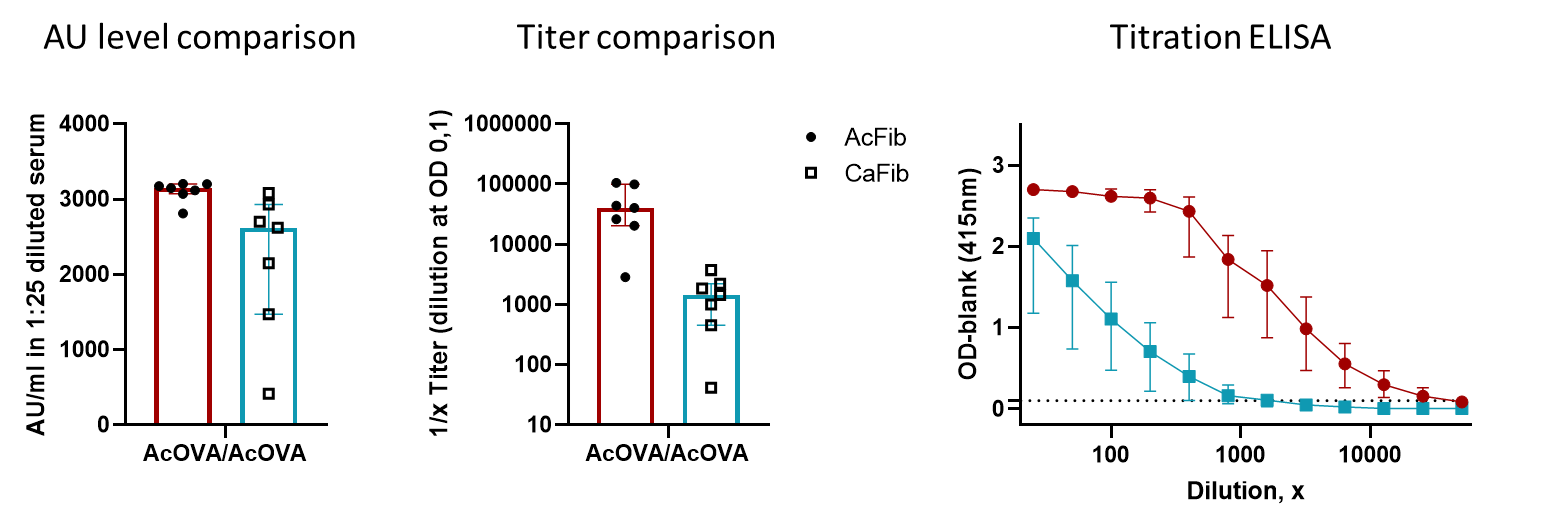


**Supplementary figure 4. Example comparing level vs titer analyses of anti-AcFib and anti-CaFib reactivities in AcOVA/AcOVA-immunized mice.**

ELISA reactivities against acetylated and carbamylated fibrinogen were analyzed either by determining arbitrary units at a fixed concentration (using one of the mice with highest signal as a standard) or by determining titers.
